# Contrastive learning with token projection for Omicron pneumonia identification from few-shot chest CT images

**DOI:** 10.3389/fmed.2024.1360143

**Published:** 2024-05-02

**Authors:** Xiaoben Jiang, Dawei Yang, Li Feng, Yu Zhu, Mingliang Wang, Yinzhou Feng, Chunxue Bai, Hao Fang

**Affiliations:** ^1^School of Information Science and Technology, East China University of Science and Technology, Shanghai, China; ^2^Department of Pulmonary and Critical Care Medicine, Zhongshan Hospital, Fudan University, Shanghai, China; ^3^Shanghai Engineering Research Center of Internet of Things for Respiratory Medicine, Shanghai, China; ^4^Department of Pulmonary and Critical Care Medicine, Zhongshan Hospital (Xiamen), Fudan University, Xiamen, Fujian, China; ^5^Department of Nursing, Zhongshan Hospital, Fudan University, Shanghai, China; ^6^Department of Anesthesiology, Zhongshan Hospital, Fudan University, Shanghai, China; ^7^Department of Anesthesiology, Shanghai Geriatric Medical Center, Shanghai, China

**Keywords:** contrastive learning, token projection, omicron pneumonia identification, random Poisson noise perturbation, chest CT images

## Abstract

**Introduction:**

Deep learning-based methods can promote and save critical time for the diagnosis of pneumonia from computed tomography (CT) images of the chest, where the methods usually rely on large amounts of labeled data to learn good visual representations. However, medical images are difficult to obtain and need to be labeled by professional radiologists.

**Methods:**

To address this issue, a novel contrastive learning model with token projection, namely CoTP, is proposed for improving the diagnostic quality of few-shot chest CT images. Specifically, (1) we utilize solely unlabeled data for fitting CoTP, along with a small number of labeled samples for fine-tuning, (2) we present a new Omicron dataset and modify the data augmentation strategy, i.e., random Poisson noise perturbation for the CT interpretation task, and (3) token projection is utilized to further improve the quality of the global visual representations.

**Results:**

The ResNet50 pre-trained by CoTP attained accuracy (ACC) of 92.35%, sensitivity (SEN) of 92.96%, precision (PRE) of 91.54%, and the area under the receiver-operating characteristics curve (AUC) of 98.90% on the presented Omicron dataset. On the contrary, the ResNet50 without pre-training achieved ACC, SEN, PRE, and AUC of 77.61, 77.90, 76.69, and 85.66%, respectively.

**Conclusion:**

Extensive experiments reveal that a model pre-trained by CoTP greatly outperforms that without pre-training. The CoTP can improve the efficacy of diagnosis and reduce the heavy workload of radiologists for screening of Omicron pneumonia.

## 1 Introduction

In the tail of February 2022, a new round of COVID-19 epidemic caused by subvariant Omicron BA. 2 and BA. 2.2 broke out in Shanghai (1). There are more than 30 mutation sites in the spike protein of the Omicron mutant, which increases the binding ability of the virus to human cells, and the infectivity is 37.5% higher than that of the Delta variant (2, 3). Until 1 June 2022, Omicron had caused 626,811 infection cases, including 568,811 asymptomatic infections, 58,000 symptomatic cases, and 588 deaths (4), which brought great crisis and challenge to social public health security (5).

**Figure 1:**
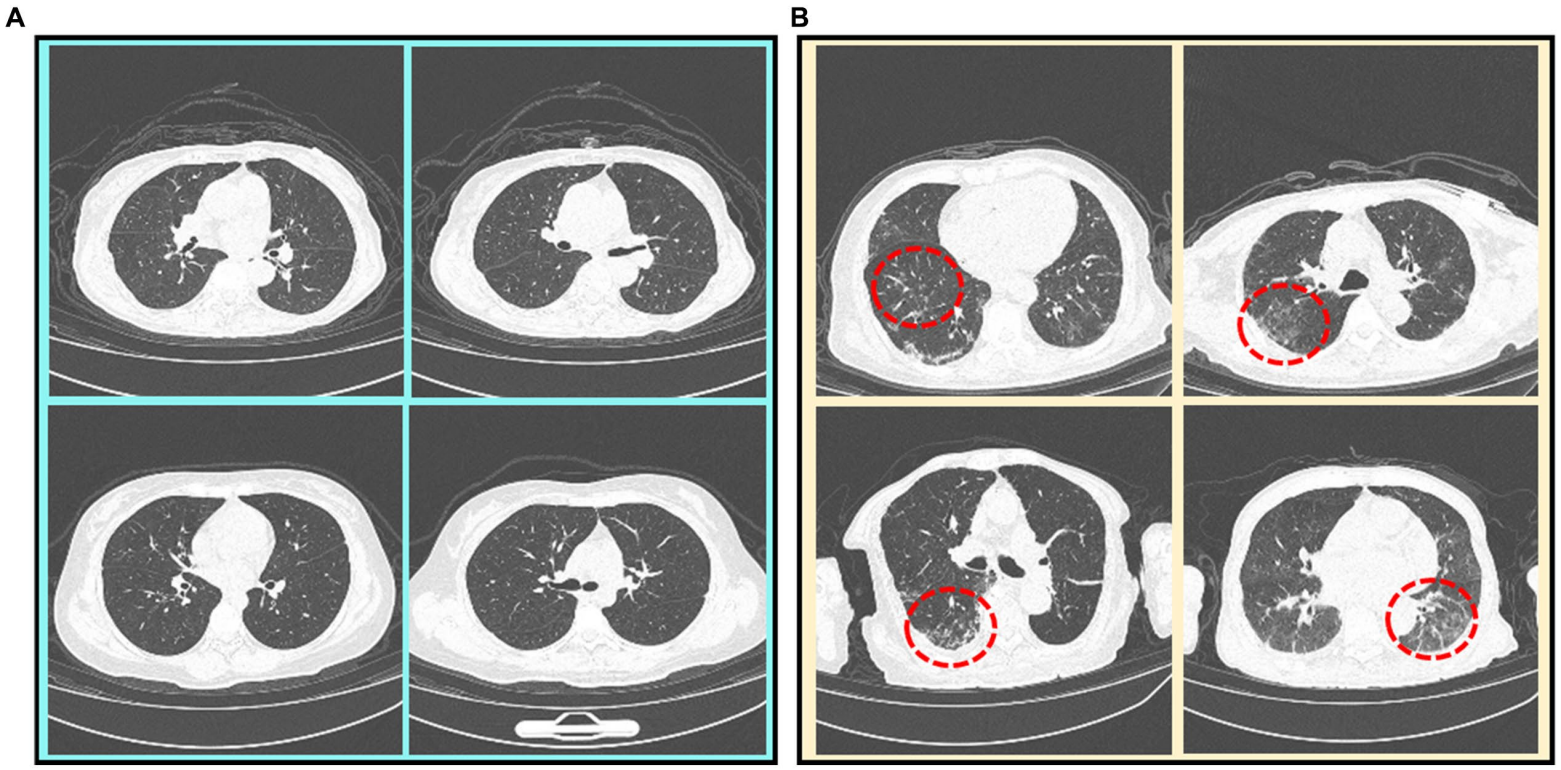
CT scan images of mild and severe Omicron pneumonia. Severe Omicron pneumonia areas are marked with red dotted circles. **(A)** Mild Omicron pneumonia. **(B)** Severe Omicron pneumonia.

Currently, the real-time reverse-transcriptase–polymerase-chain-reaction (RT-PCR) test is the main diagnostic tool (6), while chest CT imaging is increasingly recognized as a complementary or even a reliable alternative method (7, 8). Figure 1 illustrates some CT scan images of mild and severe Omicron pneumonia. All annotations have been provided by experienced doctors, who evaluate patients based on their clinical conditions and CT imaging. From that, we can find that the CT images of mild Omicron pneumonia usually show slight inflammation in the lungs. On the contrary, the CT images of severe Omicron pneumonia are more serious than those of mild Omicron pneumonia, showing more severe inflammation and damage to the lungs. Physicians need to pay more attention to patients with severe Omicron pneumonia and treat them in time. However, experienced radiologists are needed to manually identify all the thin-slice CT images (an average of 300 layers per patient) (9). This may lead to misdiagnosis due to the significantly increased workload of radiologists.

With the development of deep learning (10), researchers can extract useful information from a significant volume of annotated data (11). However, when compared to natural images, acquiring such quantities of medical data is challenging, and the annotations must be carried out by professional radiologists (12, 13). This poses huge challenges to applying deep learning to medical image analysis and processing (14). In recent years, contrastive learning methods (15–19) have achieved satisfactory results in natural image classification tasks. These methods can utilize unlabeled data to create a pre-trained model, which can then be fine-tuned with lightly annotated data for further improvement.

While some studies have investigated the effects of contrastive learning on natural image classification tasks, there remains a gap in research that specifically addresses chest CT images. The current methods based on contrast learning are insufficient in enhancing chest CT images effectively and exploring global features. To address this issue, we propose a novel contrastive learning with token projection, namely CoTP, to improve global visual representation. The token projection typically consists of a multi-head self-attention (MHSA) (20) and a fully connected (FC) layer. The MHSA can capture short and long-range visual dependencies, while the FC layer can eliminate redundant features. Moreover, we leverage the downsampling layer to reduce the cost of computation. In addition, a private Omicron dataset collected by the Geriatric Medical Center, Zhongshan Hospital, Fudan University is utilized for CoTP pre-training. Especially, data augmentations have important roles in contrastive learning methods (15). However, the widely used augmentations in contrastive learning approaches for natural images may not be suitable for chest CT. Therefore, a new data augmentation approach, random Poisson noise perturbation (PNP) is proposed for CT images, to simulate the noise in CT images. After pre-training, the feature encoder with pre-trained weights is taken out, followed by a simple max pooling and average pooling (MAP) head which can obtain different space areas occupied by objects of different categories. Then, we fine-tune the model on a sub-dataset extracted from Omicron datasets and the external SARS-CoV-2 CT-scan dataset (21), respectively. Extensive experiments reveal that a model pre-trained by the proposed CoTP greatly outperforms that without pre-training.

Our main contributions to this work are summarized as follows:

1. A novel contrastive learning with token projection, namely CoTP, is proposed to improve the diagnostic quality of few-shot Omicron chest CT images. In particular, token projection with a downsampling layer is utilized to further improve the quality of the global visual representations and reduce the computational cost. In addition, the MAP head is employed to obtain different spatial regions occupied by objects of different categories.
2. We present a new Omicron dataset approved by the institutional review board of Zhongshan Hospital, Fudan University in Shanghai. Furthermore, we leverage a new data augmentation approach, random Poisson noise perturbation (PNP) to simulate the noise in CT images which is more realistic.
3. We verify the effectiveness of the proposed CoTP on the private Omicron dataset and the external SARS-CoV-2 CT-scan dataset, which delivers promising results on both datasets.

## 2 Related work

### 2.1 Supervised learning for diagnosis of pneumonia from chest CT images

Since the outbreak of coronavirus disease COVID-19 was declared a pandemic by the WHO on 11 March, 2020, various deep learning-based methods have been implemented worldwide to promote and save critical time for pneumonia diagnosis from CT images. Wu et al. (9) proposed a multi-view fusion model to improve the efficacy of diagnosis. A previous study Mei et al. (22) designed a grad-CAM-based deep learning method for fast detection of COVID-19 cases. Another study (23) diagnosed COVID-19 via the proposed network with a multi-receptive field attention module on CT images. Moreover, Mei et al. (22) adopted ResNet (23) to rapidly diagnose COVID-19 patients using both full CT scans and non-image information. In addition, several works (24–26) also used segmentation techniques for detection. However, the current deep learning-based approaches for pneumonia diagnosis primarily rely on supervised learning, leveraging abundant labeled data to acquire precise visual representations. On the contrary, there are few-shot labeled chest CT images. Table 1 summarizes the previous studies on supervised learning for pneumonia diagnosis from chest CT images.

**Table 1:** Previous studies on supervised learning for pneumonia diagnosis from Chest CT images.

Authors	Year published	Pros.	Cons.	Results
Wu et al. (9)	2020	Axial, coronal, and sagittal views of each chest CT image are selected as the inputs of the deep learning network.	Subgroup analysis was limited by the unavailability of detailed clinical information.	81.9% AUC on CT images dataset.
Panwar et al. (27)	2020	Grad-CAM-based color visualization approach and early stopping.	Lack of ground truth boxes to detect lesions.	95% ACC on the SARS-COV-2 CT-scan dataset.
Mei et al. (22)	2020	Demographic and clinical data are also integrated by an MLP network to rapidly diagnose patients.	The study has a small sample size.	92% AUC on the COVID-19 dataset.
Chen et al. (24)	2020	Performing both classification and detection tasks simultaneously.	The inference time is slow	98.85% ACC in the internal retrospective dataset
Wang et al. (26)	2020	A novel noise-robust Dice loss function, adaptive teacher and student mechanisms.	Incorrect predictions tend to be related to noisy labels.	80.29% Dice on the COVID-19 pneumonia dataset
Ma et al. (28)	2021	Multi-receptive field attention module.	Lack of ground truth boxes to detect lesions.	99.01% AUC on the SARS-COV-2 CT-scan dataset.
Qiu et al. (25)	2021	Attentive Hierarchical Spatial Pyramid module and lightweight multi-scale learning.	Require a large amount of labeled data.	75.91% Dice on the COVID-19-CT dataset.

### 2.2 Contrastive learning in image analysis

Given the efficient visual representation ability of deep learning, contrastive learning has emerged as a promising approach for efficiently extracting accurate visual representations from unlabeled images (29). Wu et al. (16) first designed a framework that pulls away augmented views of different images (negative pair) while pulling in different augmented views of the same image (positive pair). Based on this idea, the two methods, SimCLRv1 (15) and MoCo-v1 (18) were proposed, which can greatly narrow the gap between supervised learning and unsupervised learning on downstream task performance. These methods, SimCLRv2 (17) and MoCo-v2 (19) employed the projection head to improve the ability of visual representation extraction and outperformed the supervised learning on downstream tasks.

The success of these methods motivated many researchers to introduce contrastive learning into medical image analysis. Sowrirajan et al. (13) utilized MoCo pre-training to improve the representation and transferability of chest X-ray Models. Zhang et al. (30) obtained medical visual representations according to contrastive learning with paired images and texts. In addition, the works of various researchers (30–32) employed contrastive learning for medical image segmentation. However, the existing contrastive mechanisms have scope for improvement for Omicron pneumonia diagnosis from chest CT images due to their inability to mine global features and lack of appropriate augmentations for chest CT images. We present the pros and cons of previous studies on contrastive learning in image analysis in Table 2.

**Table 2:** Previous studies on contrastive learning in image analysis.

Authors	Year published	Pros.	Cons.	Results
Wu et al. (16)	2018	Maximize the distinction between instances and non-parametric instance discrimination.	Compared to supervised models, it still has a significant gap.	54.0% ACC on ImageNet dataset.
Chen et al. (15)	2020	A learnable nonlinear transformation.	Require a huge batch size of 4,096.	61.9% ACC on ImageNet dataset.
He et al. (18)	2020	A dynamic dictionary with a queue and a moving-averaged encoder.	Requires a large number of negative samples as a queue.	60.6% ACC on ImageNet dataset.
Chen et al. (17)	2020	Unlabeled examples for refining and transferring the task-specific knowledge.	Require a huge batch size of 4,096.	66.6% ACC on ImageNet dataset.
Chen et al. (19)	2020	A learnable nonlinear transformation is added in MoCo-v1.	Requires a large number of negative samples as a queue.	67.5% ACC on ImageNet dataset.
Zhang et al. (29)	2020	Exploite naturally occurring paired descriptive text.	Require a large amount of text annotations.	91.2% ACC on the NCT-CRC-HE-100 K dataset.
Chaitanya et al. (31)	2020	Domain-specific contrasting strategies and local version of contrastive loss.	The computational complexity is heavy.	88.6% Dice on the ACDC dataset.
Sowrirajan et al. (13)	2021	MoCo-CXR Pre-training for chest X-ray Interpretation.	Lack of effective data augmentation.	81.3% AUC on the CheXpert dataset.
Zeng et al. (32)	2021	Generate contrastive data pairs based on the position of a slice in volumetric medical images.	lack of appropriate augmentations for medical images.	92.9% Dice on the ACDC dataset.
Wu et al. (33)	2022	The proposed network does not rely on large negative samples.	Lack of global visual representation.	89.4% Dice on the ACDC dataset.

## 3 Materials and methods

### 3.1 The pipeline for interpretation of CT images

**Figure 2:**
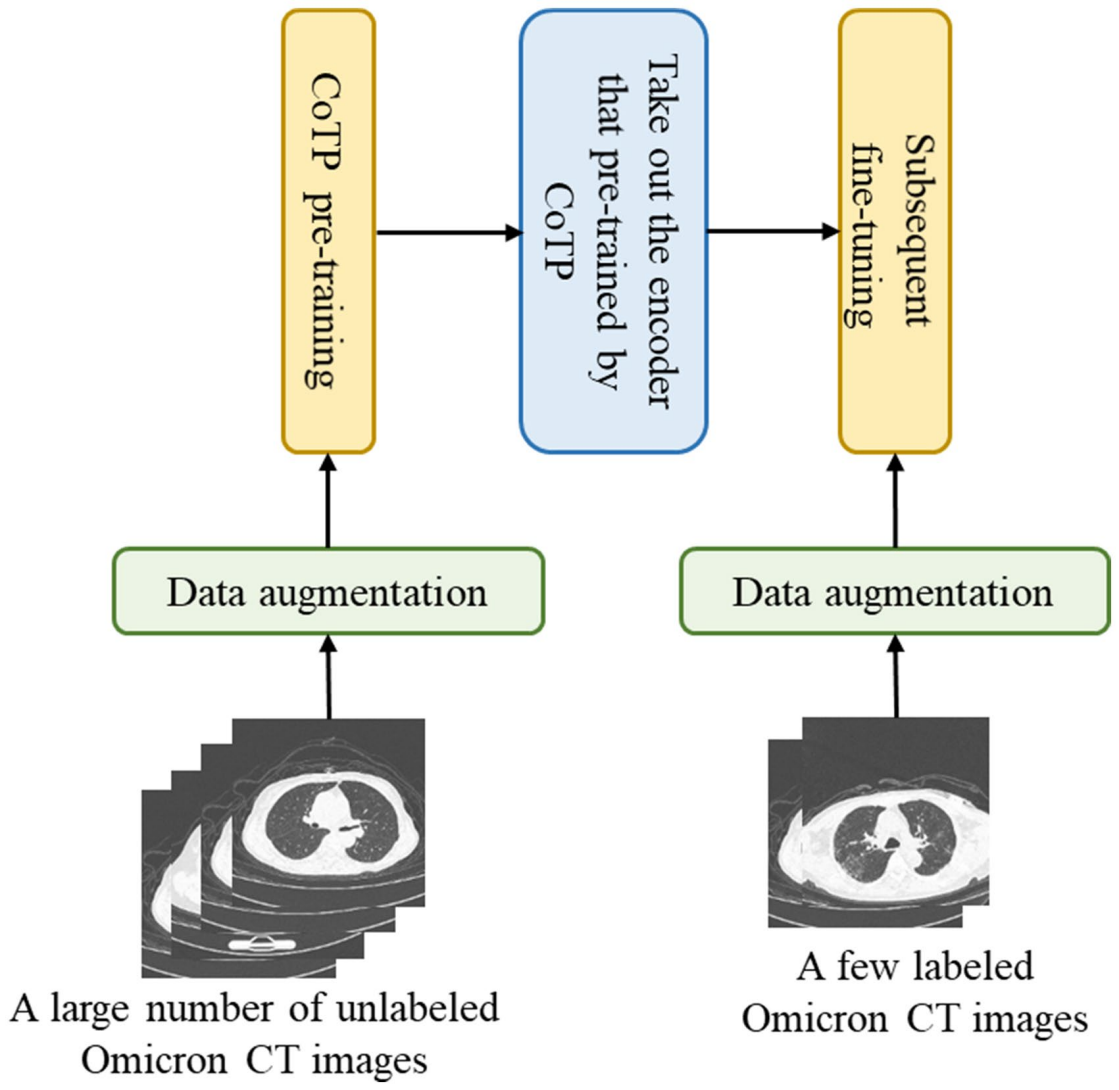
Overview of Omicron pneumonia.

The overall pipeline for CoTP pre-training and the subsequent fine-tuning with CT images are illustrated in Figure 2. There are two stages for CT image interpretation: the CoTP pre-training stage and subsequent fine-tuning. First, we converted and exported the DICOM files of Omicron patients into JPEG formats and employed CoTP to pre-train the feature encoder using unlabeled Omicron CT images. Second, the feature encoder with pre-trained weights was taken out, followed by a simple linear classifier. Then, we fine-tuned the baseline with a few labeled CT images.

### 3.2 Random Poisson noise perturbation

Data augmentation is widely used in contrastive learning and is crucial for learning good representations (15). Nevertheless, most existing natural image data augmentations may not be suitable for chest CT images. For example, random crops and cutouts may remove or mask the lesion area of CT images. Meanwhile, color jitter and random grayscale transformation are no longer applicable to grayscale CT images.

**Figure 3:**
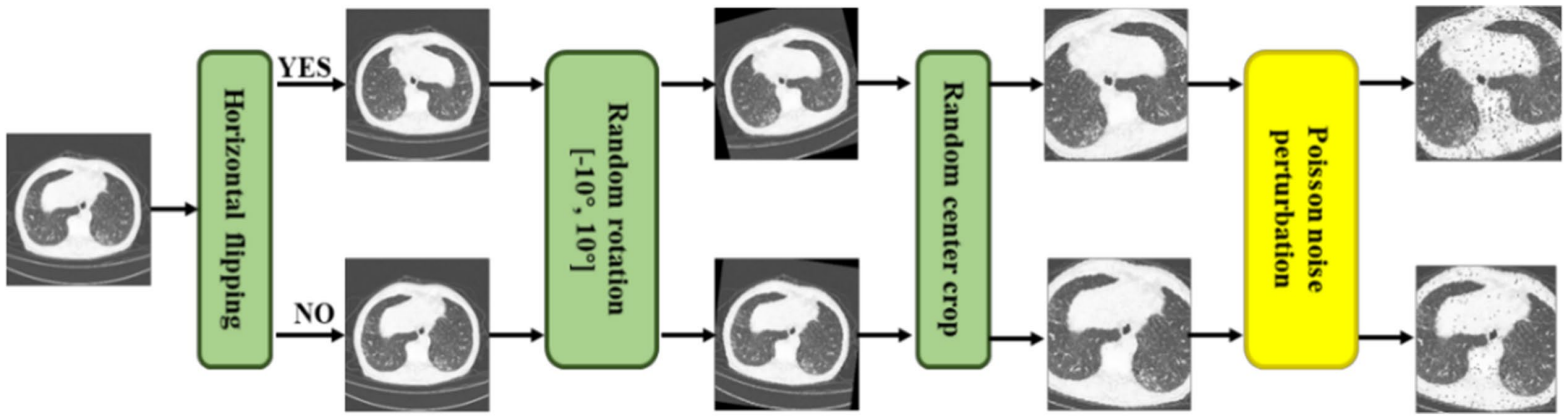
Illustrations of a series of CT augmentation methods, i.e., random horizontal flipping, random center crop, random rotation (10 degrees), and the proposed random Poisson noise perturbation.

As shown in Figure 3, we not only utilized traditional methods, i.e., random horizontal flipping, random center crop, and random rotation (10 degrees) but also a new data augmentation approach, random Poisson noise perturbation for CT images. Poisson distributed noise is a well-known data augmentation (34, 35). However, this was the first time that Poisson-distributed noise was applied to contrastive learning instead of Gaussian noise perturbation. In the process of scanning CT images, various noises will be generated due to the photoelectric interaction, and the noise distribution is more accurately characterized by the Poisson distribution (36). Consequently, we employed random Poisson noise perturbation to simulate the noise in CT images as a new data augmentation for CT images. First, we performed fan beam projection (37) transformation on the CT image X , and added Poisson noise as Eq. (1), where b stands for the number of photons. Here b is set as le-6.


(1) $$I_{n}=\mathrm{Poisson}\left(b\cdot \exp\left(-\mathrm{Fanbeam}\left(X\right)\right)\right)$$

Then, $I_{n}$ has to be processed with a logarithm and transform (iFanbeam) from the classical filtered back-projection (FBP) algorithm (38) to the image domain $X^{\prime }$ , as Eq. (2). Thus, we gained the Poisson noise CT image according to Eqs. (1, 2) as a more realistic data augmentation.

(2) $$X^{\prime }=\mathrm{iFanbeam}\left(\ln\left(b/I_{n}\right)\right)$$

### 3.3 Overview of the proposed CoTP

#### Algorithm 1 Pseudocode of CoTP.

**Table:** 

**Input:** batch size B , constant temperature τ , negative memory bank $N=\left\{n_{0,}\;n_{1,}\;n_{2,\cdot \cdots }\right\}$ , encoder networks for query and key E:q , E:k , Token projection for query and key T:q , T:k , **for** sampled minibatch ${\left\{x_{q}\right\}}_{q=1}^{B}$ do **for** all $q\in \left\{1,\ldots ,B\right\}$ **do** draw two augmentation functions CTAug1, CTAug2 # augmentation for query $V_{q}=E:q\left(q\right)$ # encoder $Z_{q}=T:q\left(f_{q}\right)$ # Token projection # augmentation for key $V_{k}=E:k\left(q\right)$ # encoder $Z_{k}=T:k\left(f_{k}\right)$ # Token projection **end for** **define** $L=-\log\left(\frac{\exp\left(Z_{q}\cdot Z_{k}/\tau \right)}{\exp\left(Z_{q}\cdot Z_{k}/\tau \right)+\sum_{i=0}^{N}\exp\left(Z_{q}\cdot n_{i}/\tau \right)}\right)$ update networks E:q and T:q to minimize L **define** momentum update: $\omega_{k}=m\omega_{k}+\left(1-m\right)\omega_{q}$ update networks E:k and T:k by momentum update **end for** **update** negative memory bank **return** encoder network E:q , and throw away E:k

Algorithm 1 summarizes the proposed CoTP.

#### 3.3.1 Feature encoder

**Figure 4:**
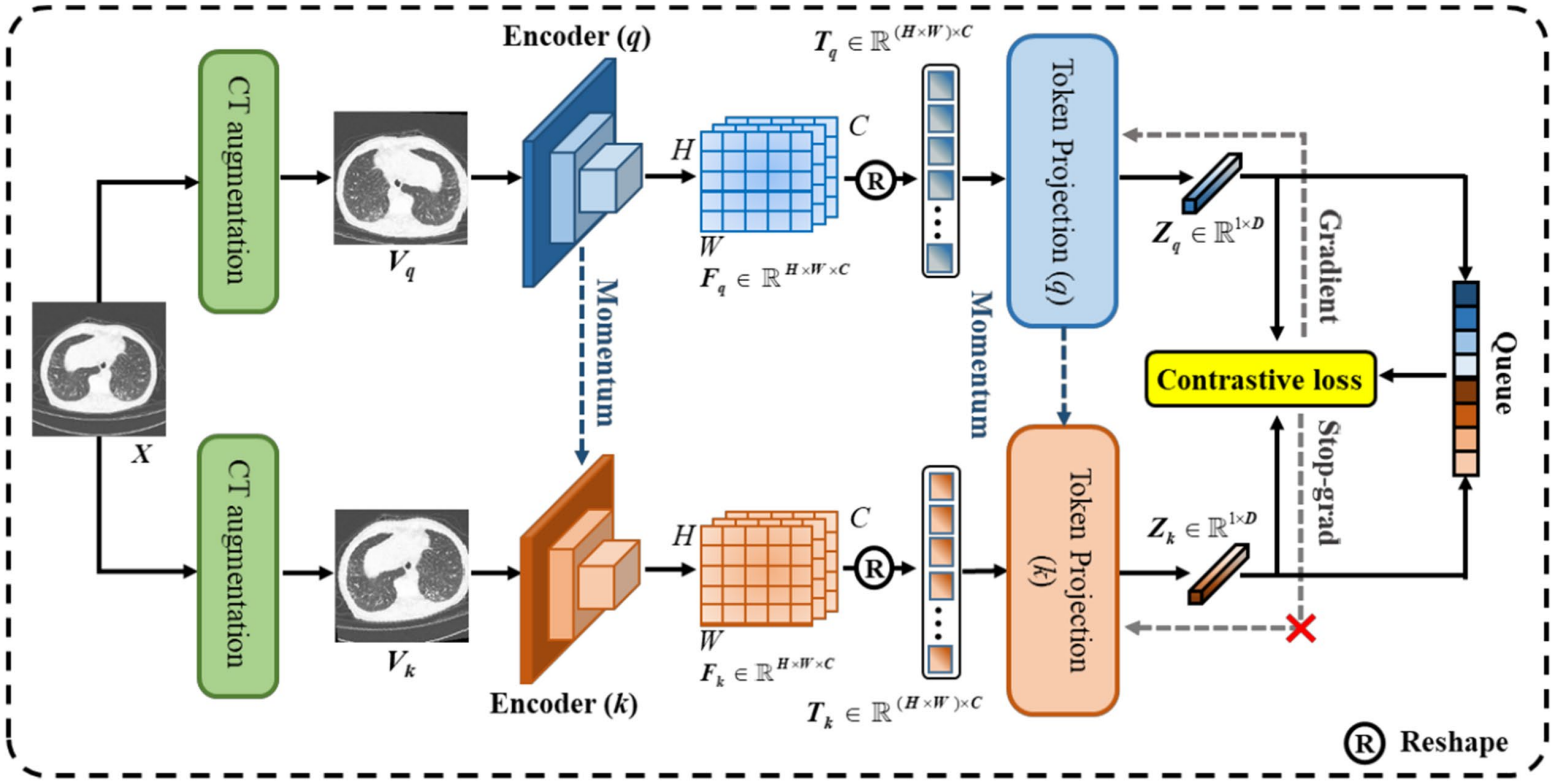
The overall architecture of the proposed CoTP.

As shown in Figure 4, we designed CoTP to learn global visual representations effectively from unlabeled CT images. Given a CT image, X , we utilized two different augmentations to create two views of the same example, $V_{q}$ and $V_{k}$ . Then, we employed ResNet50 (23) which removed the entire global pooling and Multilayer Perceptron (MLP) parts as the feature encoder. The $V_{q}$ and $V_{k}$ are mapped via encoders (*q*) and (*k*), to generate visual representations $F_{q}\in R^{H\times W\times C}$ and $F_{k}\in R^{H\times W\times C}$ , respectively. Here, H , W , and C are the length, width, and dimension of the feature map. The pseudocode of CoTP is shown in Algorithm 1.

#### 3.3.2 Token projection

**Figure 5:**
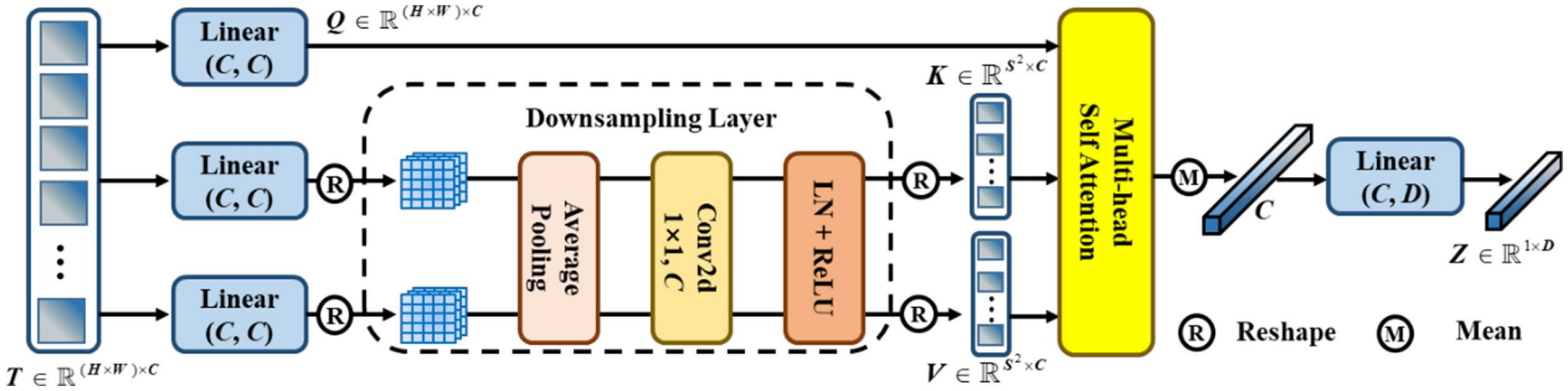
The pipeline of token projection. The downsampling layer is designed for the reduction of calculation by reducing the resolution of feature.

Traditional contrastive learning (17, 19) typically uses a global pooling operation and an MLP as a projection head to improve the visual representations. Innovatively, we designed a token projection instead of a traditional projection head, as shown in Figure 5.

To begin with, we reshaped the feature $F\in R^{H\times W\times C}$ to $T\in R^{\left(H\times W\right)\times C}$ . Then, T was passed through three different linear projections and yielded a query Q , a key K , and a value V . Furthermore, we performed average pooling with a pooling size of S for K and V to reduce the cost of computation. Here, we set S=7 . After that, a convolution with 1 kernel size and 1 stride size was utilized to fuse the feature. Then, we gained the $K\in R^{S^{2}\times C}$ and $V\in R^{S^{2}\times C}$ after performing layer normalization (LN) and ReLU activation functions. Afterward, we performed multi-head self-attention (MHSA) on Q , K , and V , as shown in Eq. (3),

(3) $$T^{\prime }=\mathrm{MHSA}\left(Q,K,V\right)=\mathrm{Softmax}\left(\frac{QK^{T}}{\sqrt{C}}\right)V$$

Then, we calculated the mean score of $T^{\prime }\in R^{\left(H\times W\right)\times C}$ along the dimension of the column. Finally, we performed a linear projection to eliminate redundant features and obtain $Z\in R^{1\times D}$ . In particular, we set the dimension D=128 .

#### 3.3.3 Update the weights

To meet a large number of negative sample pairs and reduce the computing cost of Graphics Processing Unit (GPU), a memory bank was used to store the negative samples generated by the encoder (*q*), in advance. Hence, we obtained a set of encoded (*k*) samples $E_{k}=\left\{Z_{k},n_{0,}\;n_{1,}\;n_{2,\cdot \cdots }\right\}$ . Out of all the encoded (*k*) samples in the set *E_k_* for each encoded query $Z_{q}$ , a single positive key $Z_{k}$ was matched, while the remainder of the keys (negative keys) represented different images. A contrastive loss function is represented in Eq. (4) as follows, whose value is low when $Z_{q}$ is close to its positive key $Z_{k}$ and moves away from all other encoded (*k*) samples:


(4) $$L=-\log\left(\frac{\exp\left(Z_{q}\cdot Z_{k}/\tau \right)}{\exp\left(Z_{q}\cdot Z_{k}/\tau \right)+\sum_{i=0}^{N}\exp\left(Z_{q}\cdot n_{i}/\tau \right)}\right)$$

where *τ* is a temperature hyper-para (16), and the number of negatives N is set at 32,256. We updated the weights $\omega_{q}$ of the encoder (*q*) and token projection (*q*) by back-propagation, while the weights $\omega_{k}$ of the encoder (*k*) and token projection (*k*) were updated by momentum update (18), as Eq. (5), where m is 0.999 to update the weights slowly.


(5) $$\omega_{k}=m\omega_{k}+\left(1-m\right)\omega_{q}$$

### 3.4 Subsequent fine-tuning

**Figure 6:**
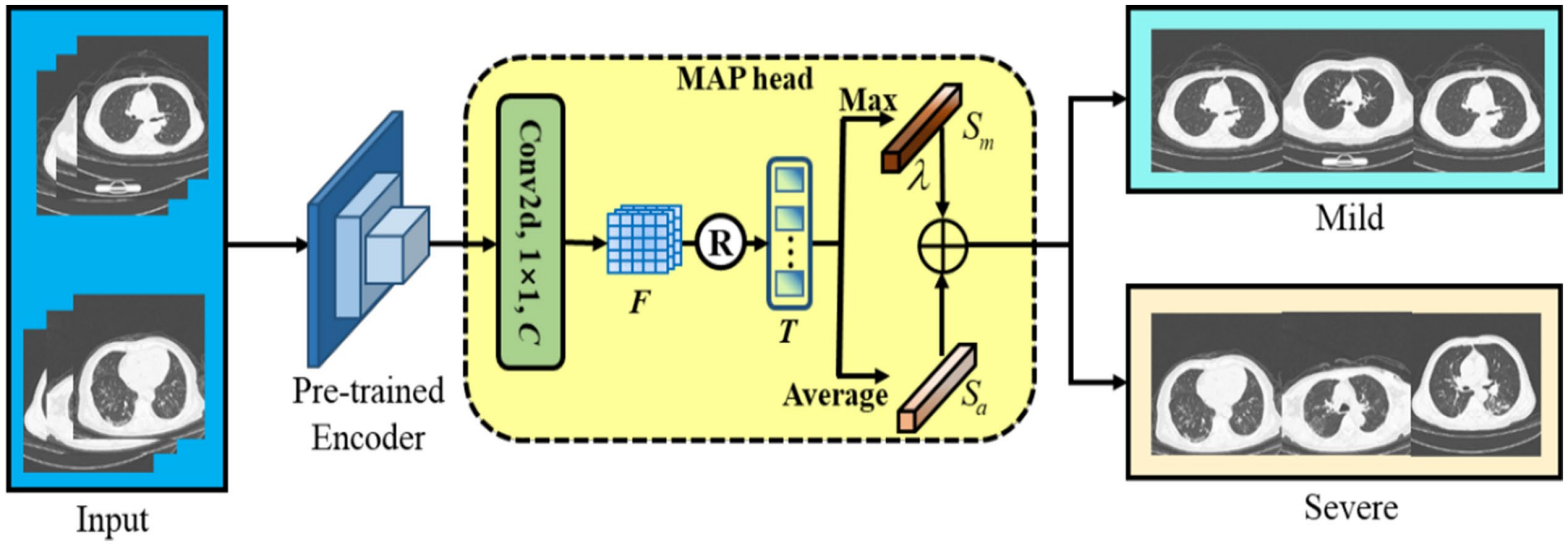
The backbone of the subsequent fine-tuning. We leverage max pooling head and average pooling head to improve classification performance.

After CoTP pre-training, we took out the feature encoder with pre-trained weights, followed by a max pooling and average pooling (MAP) head, as shown in Figure 6. First, we performed a 1×1 convolution and reshaped the feature $F\in R^{H\times W\times C}$ to $T\in R^{\left(H\times W\right)\times C}$ . Here, *C* denotes the class of categories. Afterward, we calculated the mean score and maximum score of the *T* along the dimension of the column, respectively. Finally, a Hyper-parameter λ was employed to combine the mean score $S_{a}$ and maximum score $S_{m}$ , as Eq. (6).


(6) $$S=S_{a}+\lambda *S_{m}$$

It is noteworthy that max-pooling can be considered as class-specific attention that can attain different space areas occupied by objects of different categories. In particular, we performed a simple cross-entropy loss to fine-tune the baseline with a few-shot labeled CT images.

### 3.5 Implementation details

As shown in Table 3, we utilized Python 3.7 and Pytorch 1.7.0 with PyCharm as our Integrated Development Environment (IDE), running on a PC equipped with Intel(R) i9-10940X CPU and 4 Nvidia 1,080 Ti GPUs with 48 GB memory. At the CoTP pre-training stage, Stochastic Gradient Descent (SGD) was employed as our optimizer, while the weight decay was le-4 and the momentum was 0.9. The mini-batch size refers to the number of examples (data points) that are processed together in one iteration of training in deep learning. Here, we set mini-batch size as 128, and the learning rate is initialized to 0.03. Followed by He et al. (18), we train for a total of 200 epochs, and the learning rate multiplied by 0.1 at 120 and 160 epochs.

**Table 3:** The working environment.

Hardware		Software	
CPU	GPU	IDE	Framework
Intel(R) i9-10940X	Nvidia 1,080 Ti (Numbers: 4)	PyCharm	Pytorch 1.7.0

At the subsequent fine-tuning stage, we utilized AdmW with le-3 weight decay as the optimizer. The mini-batch size refers to the number of examples (data points) that are processed together in one iteration of training in deep learning. Here, we set mini-batch size as 32, and the learning rate is initialized to le-4.

## 4 Results

The classification performances of the proposed methods were evaluated in terms of the standard metrics, such as accuracy (ACC), sensitivity (SEN), and precision (PRE) discussed in Eq. (7)–(9), where *P*, *N*, *TP*, *TN*, and *FP* denote positives, negatives, true positives, true negatives, and false positives, respectively.


(7) $$ACC=\frac{TP+TN}{N+P}$$

(8) $$SEN=\frac{TP}{P}$$

(9) $$PRE=\frac{TP}{TP+FP}$$

In addition, the mean AUC (39) was employed to evaluate the ability of the model to discriminate between different classes. Furthermore, we also used a non-parametric bootstrap (40) to estimate the variability around model performance. We performed a total of 500 bootstrap sampling with 300 cases from the test set.

### 4.1 Datasets

The study was approved by Zhongshan Hospital, Fudan University in Shanghai, China. All the chest CT scanning images in the Omicron dataset were selected from a retrospective cohort of adult Omicron patients hospitalized in Shanghai Geriatrics Center from March to July 2022. Chest CT examination was performed as part of the patient’s routine clinical care at the time of admission. The eligibility criteria were as follows: (1) having intact basic information to be retrieved (names, gender, ages, diagnosis, and severity), and (2) having CT scanning on admission. Patients with underlying lung diseases such as chronic obstructive pulmonary disease (COPD) and bronchiectasis were excluded. All patient scans were downloaded in the DICOM image format. The thickness of the CT image was 5 mm.

The diagnosis and classification of severity were based on the Diagnosis and Treatment Scheme of Pneumonia Caused by Novel Coronavirus of China (the ninth version). (National Health Commission of China. The guidelines for the diagnosis and treatment of new coronavirus pneumonia (version 9). Accessed July 25, 2023 https://www.gov.cn/xinwen/2022-06/28/content_5698168.htm). Adults were considered severe Omicron pneumonia if they met any of the following criteria: (1) tachypnea with a respiratory rate ≥ 30 breaths/min; (2) oxygen saturation (at rest) ≤ 93%; (3) PaO2/FiO2 ≤ 300 mmHg; (4) radiographic progression of more than 50% of the lesion over 24–48 h; or (5) respiratory failure, shock, or other organ failures.

Following the above standards, we retrospectively collected high-resolution CT images of 73 patients with mild Omicron pneumonia and 56 patients with severe Omicron pneumonia. The Omicron dataset and demographic characteristics of patients are detailed in Table 4. Initially, we converted and exported the DICOM files of Omicron patients into JPEG formats with 1,500 HU window width and 750 HU window level. After that, we obtained 50,500 unlabeled CT images with the size of 224×224 for CoTP pre-training.

**Table 4:** Demographics and baseline characteristics of patients in the Omicron dataset.

	Age		Gender	
	< 60 years (16–58)	≥ 60 years (60–96)	Male	Female
Mild Omicron pneumonia	28	45	38	35
Severe Omicron pneumonia	3	53	34	22

**Figure 7:**
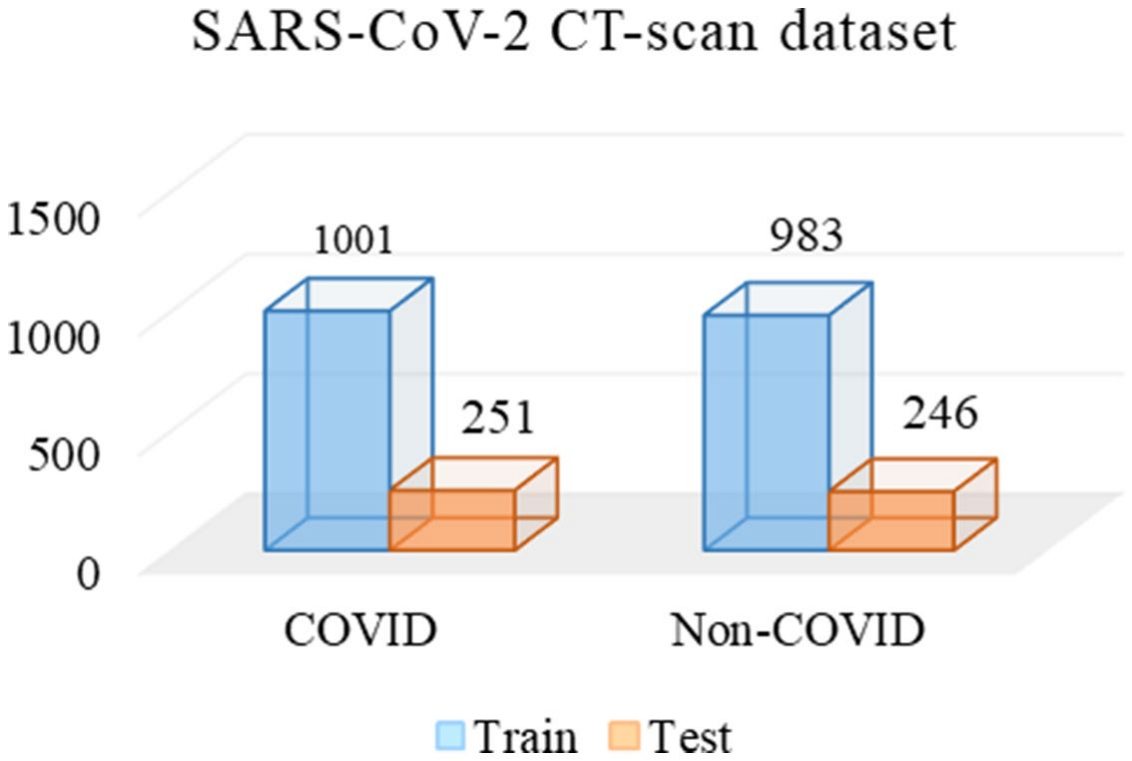
Distribution of training and testing set in SARS-CoV-2 CT-scan dataset.

Two experienced radiologists were selected, and they first labeled 2,742 CT images from the Omicron dataset. Then, we used the remaining data for CoTP pre-training. Note that the labeled CT images were excluded from the CoTP pre-training. The distribution of training and testing set in the Omicron dataset is shown in Table 5. In addition, we utilize the external SARS-CoV-2 CT-scan dataset presented by Soares et al. (21) to evaluate the transferability of CoTP. As shown in Figure 7, 1,252 CT scans were positive for SARS-CoV-2 infection (COVID-19), while 1,229 CT scanning for patients non-infected by SARS-CoV-2.

**Table 5:** Distribution of training and testing set in Omicron dataset.

	Mild Omicron pneumonia (n images)	Severe Omicron pneumonia (n images)	Total
Pre-training	–	–	129 (50, 500)
Training set	58 (1302)	45 (904)	103 (2206)
Testing set	15 (330)	11 (206)	26 (536)

### 4.2 Transfer performance of CoTP representations for omicron pneumonia diagnosis

**Figure 8:**
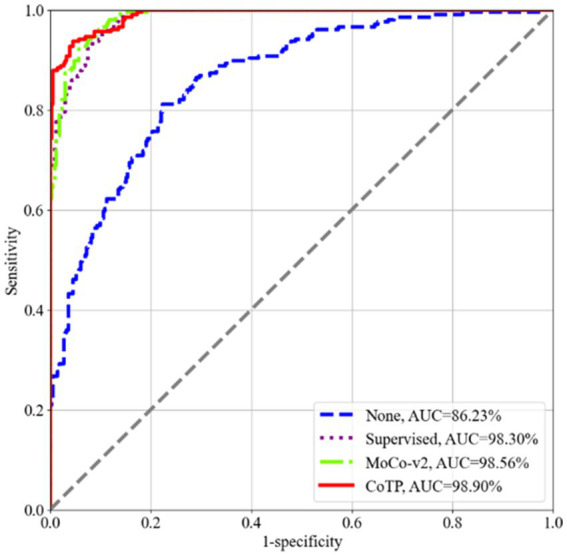
ROC curves of the four types of pre-training methods using ResNet50 on the Omicron dataset. Our CoTP achieves the highest 98.90% AUC.

To assess the effectiveness of the visual representations extracted by CoTP, we employed VGG16 (41), DenseNet121 (42), and ResNet50 (23), as our backbones and selected six types of pre-training methods for comparison. As depicted in Table 6, the non-pre-training method’s weights were randomly initialized, while the supervised pre-training method underwent pre-training on ImageNet-1k (43). In addition, we presented a more comprehensive comparison with the existing contrastive methods, such as SimCLRv1 (15), MoCo-v1 (18), SimCLRv2 (17), and MoCo-v2 (19) to prove the effectiveness of the proposed CoTP method. We evaluated the classification performance of the model using mean AUC, accuracy (ACC), sensitivity (SEN), and precision (PRE) of each infection type. Based on Table 6 and Figure 8 we gained the following observations: (1) Pre-training method plays an important role in improving model performance. The ResNet50 with supervised learning on ImageNet-1 k can achieve more 8.02% ACC, 10.43% SEN, and 9.71% PRE than that without pre-training. (2) Our CoTP pre-training method outperforms the supervised method and the contrastive learning methods, which gains 83.54, 91.32, and 92.35% ACC by VGG16, DenseNet121, and ResNet50, respectively. (3) Our CoTP achieves more 8.07, 7.37, 4.11 and 2.56% AUC than the SimCLRv1 (15), MoCo-v1 (18), SimCLRv2 (17), and MoCo-v2 (19) by ResNet50, respectively.

**Table 6:** The transfer results of Omicron pneumonia diagnosis.

Architectures	Pre-training		ACC (%)	SEN (%)	PRE(%)
	Method	Dataset			
VGG16 (41)	None	None	73.28	73.39	72.18
	supervised	ImageNet-1 K	80.19	80.66	79.83
	SimCLRv1 (15)	Omicron	79.79	79.16	78.75
	MoCo-v1 (18)	Omicron	79.82	79.24	78.93
	SimCLRv2 (17)	Omicron	80.24	80.98	80.28
	MoCo-v2 (19)	Omicron	81.06	81.27	82.56
	CoTP	Omicron	**83.54**	**86.02**	**84.13**
DenseNet121 (42)	None	None	76.73	76.92	76.77
	supervised	ImageNet-1 K	88.06	88.80	88.14
	SimCLRv1 (15)	Omicron	86.89	87.49	87.20
	MoCo-v1 (18)	Omicron	87.18	87.68	87.33
	SimCLRv2 (17)	Omicron	88.24	80.98	80.28
	MoCo-v2 (19)	Omicron	88.30	88.97	88.36
	CoTP	Omicron	**91.32**	**92.01**	**90.92**
ResNet50 (23)	None	None	77.61	77.90	76.69
	supervised	ImageNet-1 K	85.63	88.33	86.40
	SimCLRv1 (15)	Omicron	84.28	87.49	85.17
	MoCo-v1 (18)	Omicron	84.98	87.73	85.67
	SimCLRv2 (17)	Omicron	88.24	88.98	87.28
	MoCo-v2 (19)	Omicron	89.79	89.51	88.69
	CoTP	Omicron	**92.35**	**92.96**	**91.54**

The highest scores are shown in boldface.

**Figure 9:**
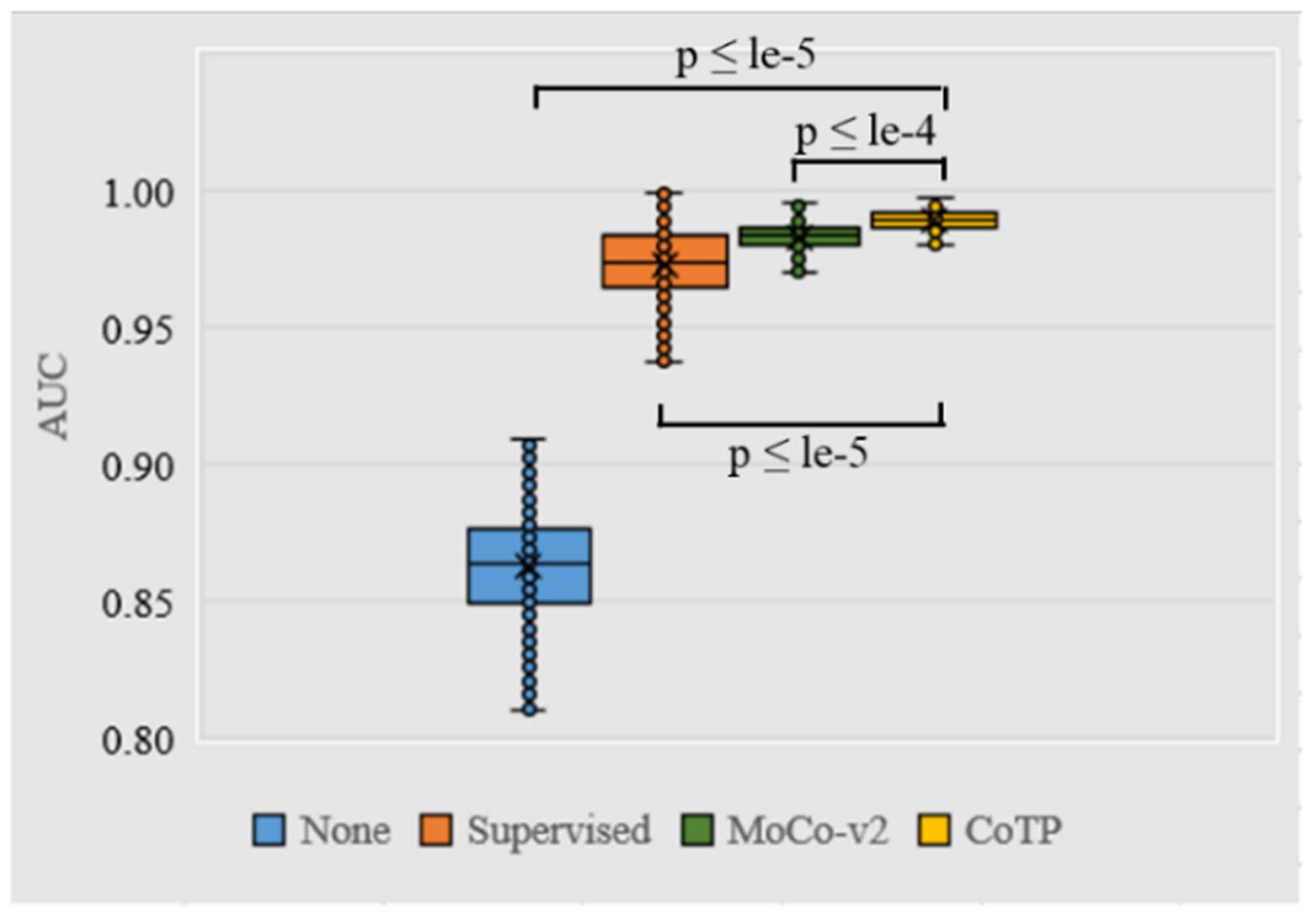
Box plot of AUC values produced by different pre-training methods using ResNet50 based on the Omicron dataset. We use 500 bootstrap samples with 300 cases to calculate the AUC which can evaluate the robustness of the model.

As shown in Figure 9, we measured the dispersion of the test set using a box plot and performed statistical significance testing using a paired t-test. From this, we observed that CoTP significantly outperforms the non-pre-training method (*p*-value < le-5) and the supervised method (*p*-value < le-5), while performing slightly better than contrastive learning MoCo-v2 (19) (*p*-value < le-4). Moreover, it can be seen that our CoTP method had better robustness than other types of pre-training methods since the distribution of AUCs was more concentrated.

### 4.3 Transfer benefit of CoTP pre-training on an external SARS-CoV-2 CT-scan dataset

**Figure 10:**
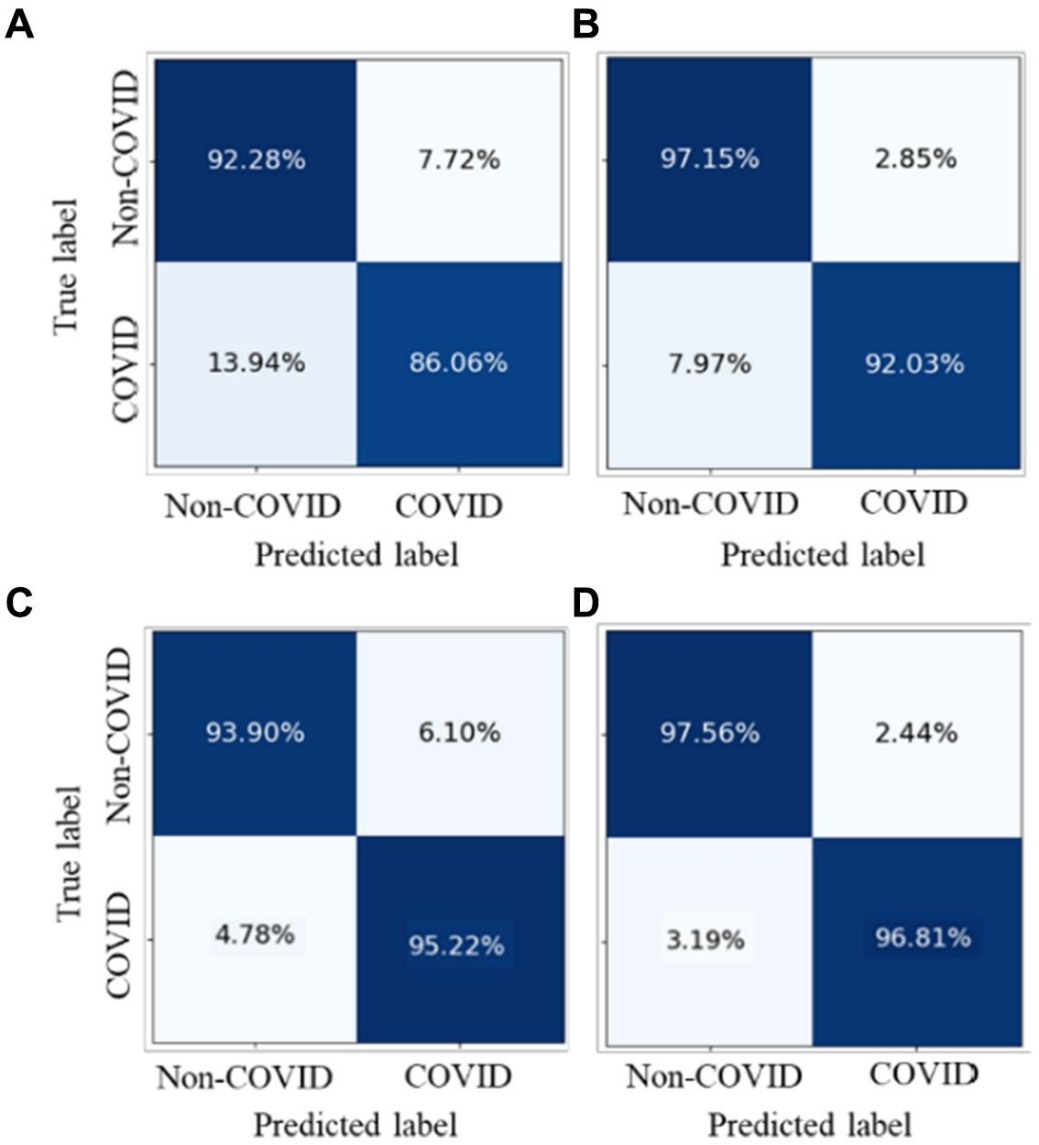
Comparison of the performance of four types of pre-training methods in identifying COVID-19 pneumonia from CXR images.

We conducted experiments to test whether CoTP pre-trained chest CT representations acquired from a source dataset (Omicron dataset) transfer to an external dataset, the SARS-CoV-2 CT-scan dataset. Table 8 demonstrates the classification results of the previous methods (27, 28, 44–50) and six types of pre-training methods based on ResNet50, while the confusion matrices for four of them are shown in Figure 10. Based on these various experimental results, we can draw the following conclusions: (1) Visual representations learned from CoTP have better transferability in downstream tasks than those from ImageNet pre-training. (2) By taking advantage of the ability of our CoTP pre-training, our model outperforms all other contrastive methods on all metrics with a large margin in discriminating between COVID and non-COVID from CT images. For example, the ACC score of CoTP increased by 7.25 and 1.01% comparing the non-pre-training and MoCo-v2 pre-training, respectively.

**Table 7:** Statistical significance testing for age and gender in the Omicron dataset.

	Total	Mild	Severe	$\chi^{2}$	*p*
Age < 60	31	28 (90.3%)	3 (9.7%)	18.902	< le-3
Age ≥ 60	98	45 (45.9%)	53 (54.1%)		
Male	72	38 (52.8%)	34 (47.2%)	0.331	0.565
Female	57	35 (61.4%)	22 (38.6%)		

The chi-square test is used to calculate the statistical significance of age and gender.

**Table 8:** The performance of MoCo-TP pre-training on the SARS-CoV-2 CT-scan dataset.

Architectures	Pre-training		ACC (%)	AUC (%)
	Method	Dataset		
Pramod et al. (44)	supervised	ImageNet-1 K	85.5	96.6
Even et al. (45)	supervised	ImageNet-1 K	86.6	86.09
Yang et al. (46)	supervised	ImageNet-1 K	89	-
Ahmed et al. (47)	supervised	ImageNet-1 K	90.8	90
Pradeep et al. (48)	supervised	ImageNet-1 K	-	98
Wang et al. (49)	Contrastive	ImageNet-1 K	90.83	96.24
Patel et al. (50)	Wavelet transform	None	93.4	93.62
Harsh et al. (27)	supervised	ImageNet-1 K	95	95
Ma et al. (28)	supervised	ImageNet-1 K	95.16	99.01
ResNet50 (23)	None	None	89.13	95.71 (95.65–95.78) *
	supervised	ImageNet-1 K	94.57	98.81 (98.78–98.84) *
	SimCLRv1 (15)	Omicron	93.78	97.82 (97.61–98.03) *
	MoCo-v1 (18)	Omicron	94.20	98.56 (98.42–98.70) *
	SimCLRv2 (17)	Omicron	95.06	98.96 (98.80–99.02) *
	MoCo-v2 (19)	Omicron	95.37	99.26 (98.75–99.29) *
	CoTP	Omicron	**96.58**	99.79 (99.78–99.80) *

The highest scores are shown in boldface.

*Quantitative data were presented as values (95% confidence interval).

## 5 Discussion

Recently, contrastive learning methods have achieved satisfactory results on natural image classification tasks, which can leverage unlabeled data to generate a pre-trained model. However, the existing contrastive mechanisms have scope for improvement for Omicron pneumonia diagnosis from chest CT images due to their inability to mine global features and lack of appropriate augmentations for chest CT images. Therefore, we proposed a novel contrastive learning with token projection, namely CoTP, for improving global visual representation. Furthermore, we leveraged a new data augmentation approach, random Poisson noise perturbation (PNP) to simulate the noise in CT images which is more realistic. In this section, we designed comprehensive ablation studies to assess the effectiveness of each component in the CoTP network.

### 5.1 Statistical significance testing for baseline characteristics of patients

First, we utilized the chi-square test for statistical significance test for baseline characteristics of patients, including age and gender. Based on Table 7, we can see that there is no significant difference in gender (*p* = 0.597), while age shows a statistical significance (*p* = 0.000). It is noted that we need to pay more attention to elderly patients since they are more vulnerable to severe Omicron pneumonia.

### 5.2 Effects of using different encoders on CoTP performance

**Figure 11:**
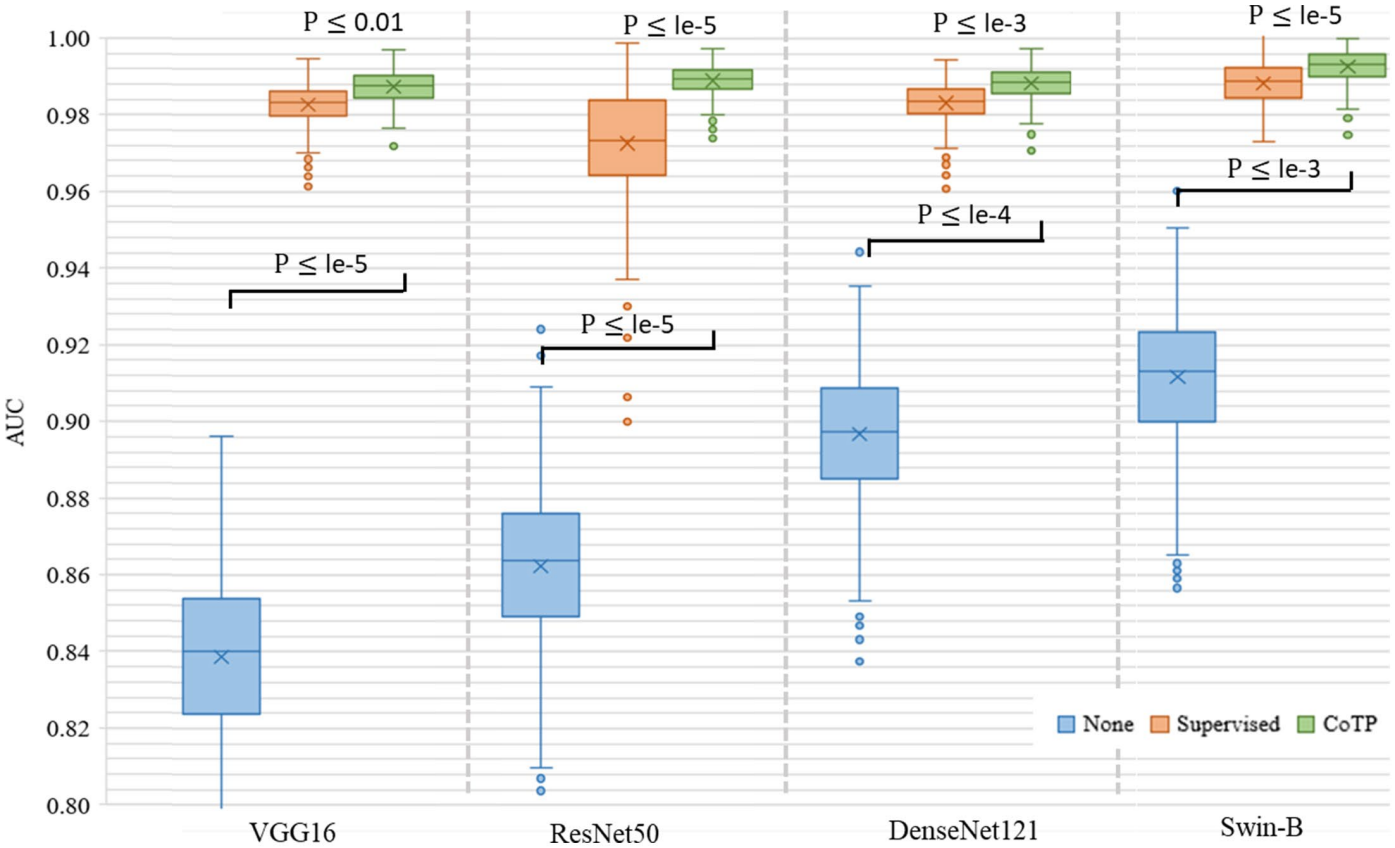
Box plot of AUC values produced by different encoders and types of pre-training methods based on the Omicron dataset. We use 500 bootstrap samples with 300 cases to calculate the AUC which can evaluate the robustness of the model.

Then, we leveraged several encoders VGG16 (41), ResNet50 (23), DenseNet121 (42), and Swin-B (51) for performance comparison, as shown in Figure 11. The results indicated that both convolutional neural networks (CNN)-based encoders and transformer-based encoders exhibited higher AUC and better robustness after pre-training with CoTP. Moreover, it can be seen that there are significant differences between pre-training methods (i.e., P ≤ le-5 between supervised pre-training method and CoTP when using ResNet50 as a backbone).

### 5.3 Impact of the random Poisson noise perturbation on CoTP performance

In addition, we investigated the impact of the proposed Poisson noise perturbation (PNP) during the data augmentation process. Therefore, we compared the model performance with and without the PNP and also compared it with the random Gaussian noise perturbation (GNP). From Table 9, we found that PNP affected the performance. For example, the VGG16 (41) with PNP could achieve an accuracy of 0.79%, a sensitivity of 0.84, and a precision of 0.88%, which are higher than those without PNP. On the contrary, GNP could not significantly improve the performance of the model. The PNP could simulate the noise CT images, which can improve the generalization of the model.

**Table 9:** Impact of the random Poisson noise perturbation on model performance based on the Omicron dataset.

		VGG16 (41)			ResNet50 (23)			DenseNet121 (42)	
	ACC	SEN	PRE	ACC	SEN	PRE	ACC	SEN	PRE
w/o PNP	82.79	85.18	83.29	91.27	92.23	90.95	90.78	91.22	90.09
GNP	82.84	85.12	83.22	91.36	92.10	90.62	90.74	91.13	89.92
PNP	**83.58**	**86.02**	**84.17**	**92.35**	**92.96**	**91.54**	**91.36**	**92.09**	**90.96**

The highest scores in each model are shown in boldface.

### 5.4 Impact of the MAP head on subsequent fine-tuning performance

**Figure 12:**
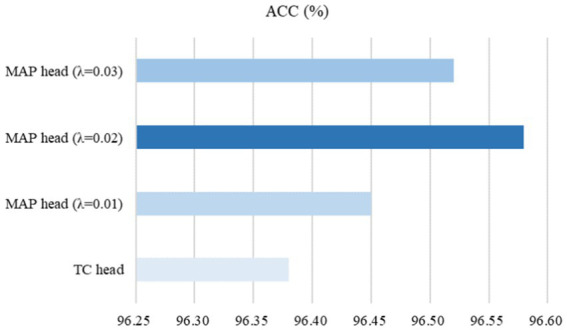
Effect of the MAP head on subsequent fine-tuning performance.

To evaluate the ability of the MAP head on subsequent fine-tuning performance, the traditional classification (TC) head, which typically consists of a global pooling operation and a fully connected layer, was used for comparative experiments on the SARS-CoV-2 CT-scan dataset. Here, we used ResNet50 (23) pre-trained by our CoTP as the backbone. Based on Figure 12, we found that the proposed MAP head outperforms the TC head and achieved the best overall performance with λ = 0.02.

### 5.5 Impact of the training data size

**Figure 13:**
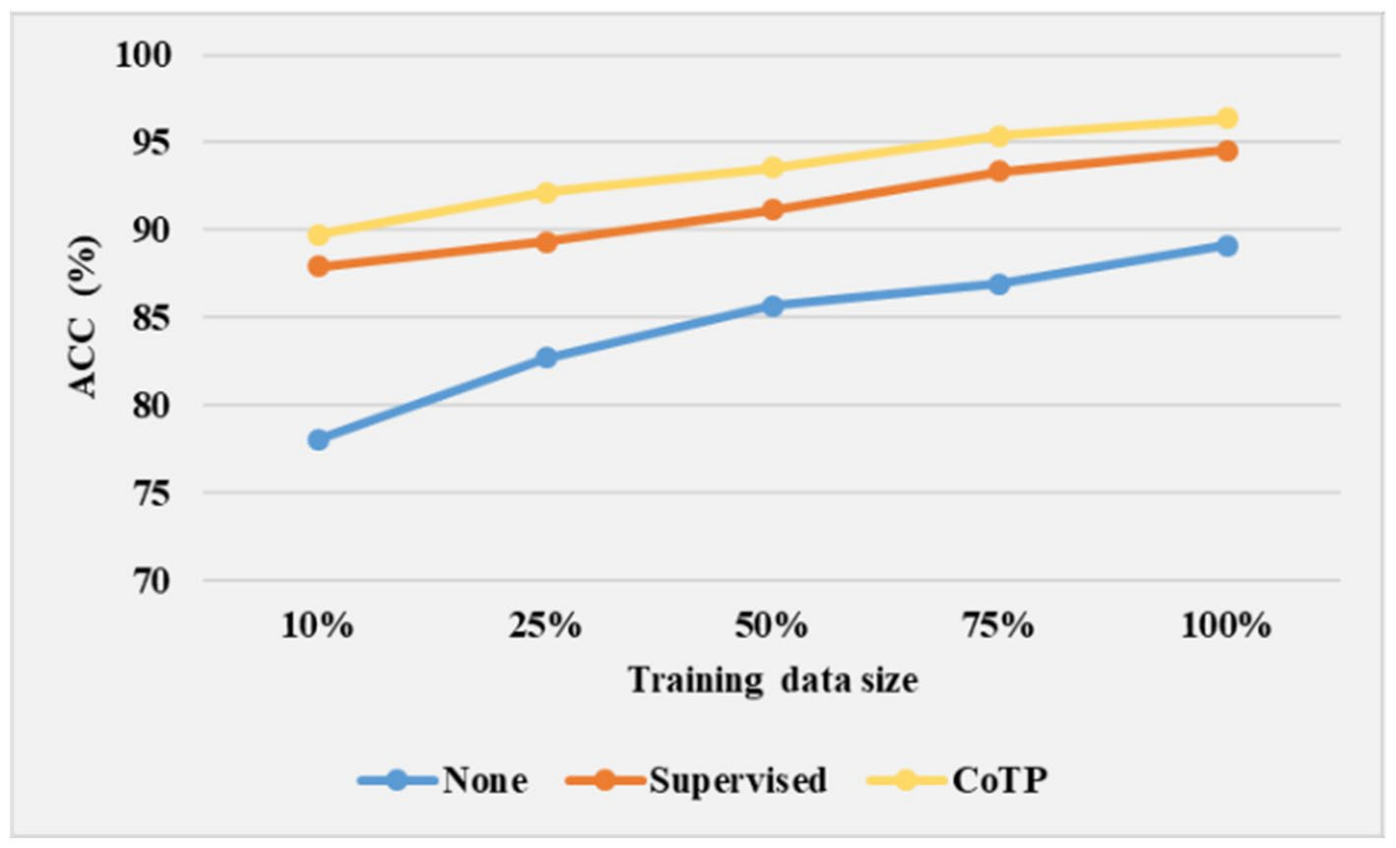
Effect of training data size on model performance.

To study the transferable ability of the model under limited labels during the fine-tuning phase, we experimented with 10, 25, 50, 75, and 100% training data size on the SARS-CoV-2 CT-scan dataset. As shown in Figure 13, we illustrated three types of pre-training methods based on ResNet50 (23). The weight of none pre-training method was randomly initialized, and the supervised pre-training method was pre-trained on ImageNet. From the results, it can be inferred that the expected trend of improving ACC is with an increase in labeled data for the fine-tuning phase. Moreover, it is promising to observe that even with a 50% training data size, the CoTP asymptotically approaches the fully supervised (100% training data size) setup.

### 5.6 Visualization of grad-CAM heat map

**Figure 14:**
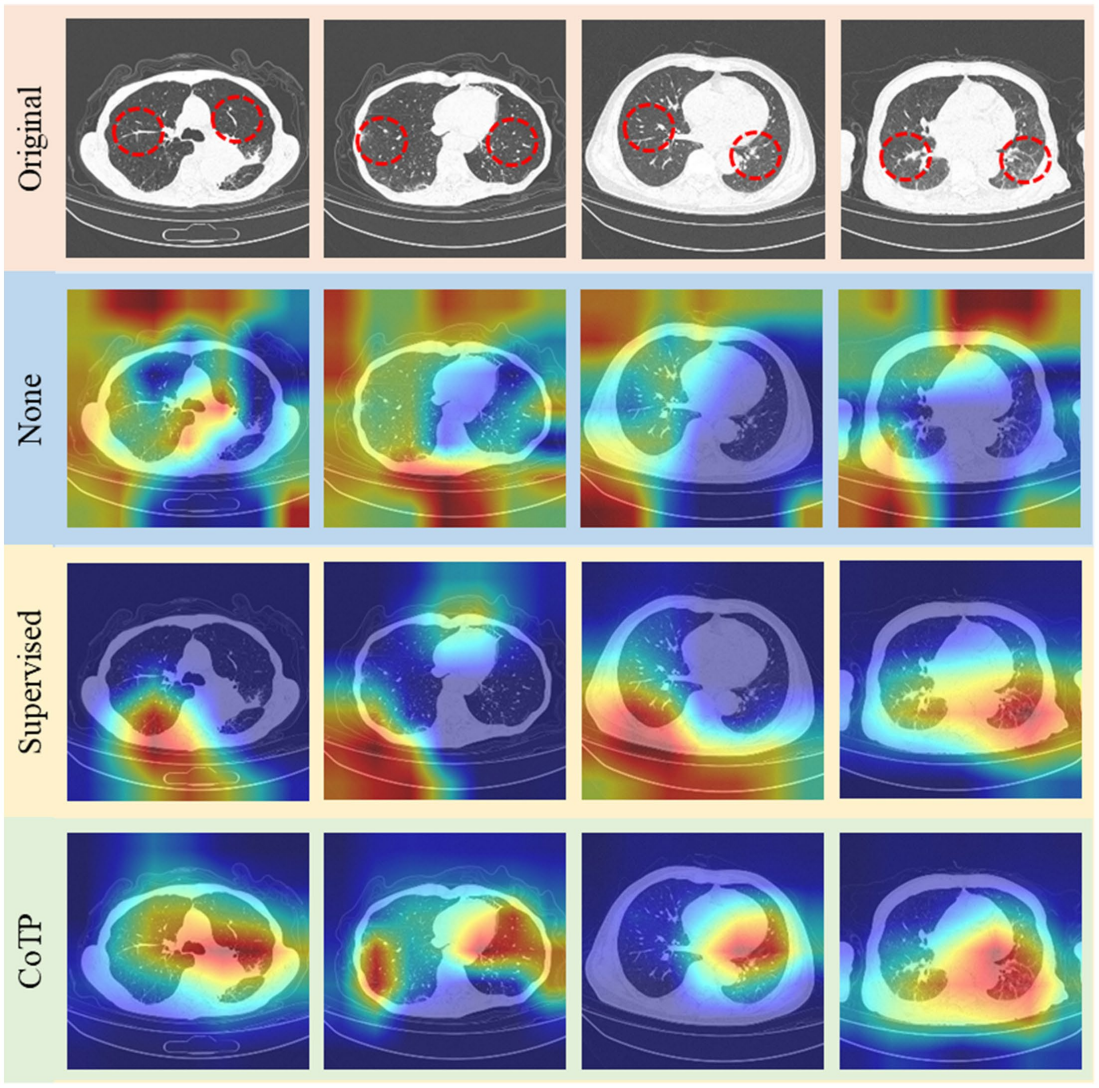
Grad-CAM visualizations of the features learned by different methods. The top row shows the original image set, followed by non-pre-training, pre-training with ImageNet, and CoTP.

Finally, we illustrated Grad-CAM (52) visualizations of the features learned by different pre-training methods based on ResNet50 in Figure 14. The higher response was highlighted in red while the lower one was demonstrated in blue. The expert annotation of the infected regions was indicated by a red dotted circle. As can be seen, the heatmaps generated by non-pre-training method are fuzzy and blurred. In addition, the heatmaps yielded by the supervised pre-training method focused on the edge areas of CT images. On the contrary, our CoTP learned more features that focus on the infection region, which can improve the classification accuracy in comparison with approaches.

### 5.7 Comparison of inference efficiency

To assess the inference efficiency, we calculated the pre-training time and parameters of MoCo-v1 (18), SimCLRv1 (15), MoCo-v2 (19), and our CoTP on the Omicron dataset. As shown in Table 10, we obtained the following observations: (1) The parameters of the methods are nearly identical. MoCo-v2 (19) adds a simple linear projection based on ResNet50 (23). Meanwhile, our CoTP included an efficient token projection in addition to ResNet50 (23). (2) The training time of SimCLRv1 (15) is the shortest among the methods because it does not utilize a memory bank. (3) Although our CoTP slightly exceeds other methods in terms of training time and parameters, it achieves the highest accuracy of 92.35% and significantly outperforms the other methods.

**Table 10:** Comparison of model efficiency on the Omicron dataset.

Method	Architecture	Training time (h)	Para (M)	ACC
MoCo-v1 (18)	ResNet50 (23)	7.8	24.03	84.98
SimCLRv1 (15)	ResNet50 (23)	6.4	24.03	84.28
MoCo-v2 (19)	ResNet50 (23) + Linear projection	7.9	24.10	89.79
CoTP (Ours)	ResNet50 (23) + Token projection	8.1	24.23	**92.35**

The highest scores in each model are shown in boldface.

## 6 Conclusion

The existing contrastive mechanisms have scope for improvement for Omicron pneumonia diagnosis from chest CT images due to their inability to mine global features and lack of appropriate augmentations for chest CT images. Therefore, we proposed a novel contrastive learning model with token projection, namely CoTP, for improving few-shot Omicron chest CT image diagnostic quality. Specifically, we designed the token projection to extract the global visual representation from unlabeled CT images. Furthermore, we leveraged random Poisson noise perturbation to simulate the noise CT images as a novel data augmentation. In addition, the MAP head which can obtain different spatial regions occupied by objects of different categories was employed to improve classification performance for subsequent fine-tuning. Extensive experiments on collected datasets demonstrated that our CoTP can provide high-quality representations and transferable initializations for CT image interpretation. In the future, we plan to design more effective pretext tasks and apply the proposed method to more medical image analysis tasks. For image segmentation and edge detection, we can employ the pre-trained encoder as a feature extraction, and then add a segmentation head or a detection head.

## Data availability statement

The data analyzed in this study is subject to the following licenses/restrictions: The SARS-CoV-2 CT-scan dataset (21) is available online. Omicron data that support the findings of this study are available from the corresponding author, YZ, upon reasonable request. Requests to access these datasets should be directed to zhuyu@ecust.edu.cn.

## Author contributions

XJ: Writing – review & editing, Writing – original draft, Software. DY: Writing – review & editing, Supervision. LF: Writing – review & editing, Data curation. YZ: Writing – review & editing, Writing – original draft, Visualization, Validation, Methodology. MW: Writing – review & editing, Formal analysis, Data curation. YF: Writing – review & editing, Resources, Data curation. CB: Writing – review & editing, Supervision, Resources. HF: Writing – review & editing, Visualization, Supervision.

## References

[1] Chen Z Deng X Fang L Sun K Wu Y Che T. Epidemiological characteristics and transmission dynamics of the outbreak caused by the SARS-CoV-2 omicron variant in Shanghai, China: a descriptive study. Lancet Reg Health-Western Pac. (2022) 29:100592. doi: 10.1016/j.lanwpc.2022.100592, PMID: 36090701 PMC9448412

[2] Li J Lai S Gao GF Shi W. The emergence, genomic diversity and global spread of SARS-CoV-2. Nature. (2021) 600:408–18. doi: 10.1038/s41586-021-04188-6 34880490

[3] Tian D Sun Y Xu H Ye Q. The emergence and epidemic characteristics of the highly mutated SARS-CoV-2 omicron variant. J Med Virol. (2022) 94:2376–83. doi: 10.1002/jmv.27643, PMID: 35118687 PMC9015498

[4] Chen X Yan X Sun K Zheng N Sun R Zhou J. Estimation of disease burden and clinical severity of COVID-19 caused by omicron BA. 2 in Shanghai, February-June 2022. Emerg Microb Infect. (2022) 11:2800–7. doi: 10.1101/2022.07.11.22277504, PMID: 36205530 PMC9683067

[5] Wilder-Smith A Freedman DO. Isolation, quarantine, social distancing and community containment: pivotal role for old-style public health measures in the novel coronavirus (2019-nCoV) outbreak. J Travel Med. (2020) 27:1–4. doi: 10.1093/jtm/taaa020, PMID: 32052841 PMC7107565

[6] Van Elden LJ Anton MAM Van Alphen F Hendriksen KA Hoepelman AI Van Kraaij MG. Frequent detection of human coronaviruses in clinical specimens from patients with respiratory tract infection by use of a novel real-time reverse-transcriptase polymerase chain reaction. J Infect Dis. (2004) 189:652–7. doi: 10.1086/381207, PMID: 14767819 PMC7110206

[7] Ai T Yang Z Hou H Zhan C Chen C Lv W. Correlation of chest CT and RT-PCR testing in coronavirus disease 2019 (COVID-19) in China: a report of 1014 cases. Radiology. (2020) 296:E32–40. doi: 10.1148/radiol.2020200642, PMID: 32101510 PMC7233399

[8] Yang Q Liu Q Xu H Lu H Liu S Li H. Imaging of coronavirus disease 2019: a Chinese expert consensus statement. Eur J Radiol. (2020) 127:109008. doi: 10.1016/j.ejrad.2020.109008, PMID: 32335426 PMC7165105

[9] Wu X Hui H Niu M Li L Wang L He B. Deep learning-based multi-view fusion model for screening 2019 novel coronavirus pneumonia: a multicentre study. Eur J Radiol. (2020) 128:109041. doi: 10.1016/j.ejrad.2020.109041, PMID: 32408222 PMC7198437

[10] LeCun Y Bengio Y Hinton G. Deep learning. Nature. (2015) 521:436–44. doi: 10.1038/nature14539 26017442

[11] Kavakiotis I Tsave O Salifoglou A Maglaveras N Vlahavas I Chouvarda I. Machine learning and data mining methods in diabetes research. Comput Struct Biotechnol J. (2017) 15:104–16. doi: 10.1016/j.csbj.2016.12.005, PMID: 28138367 PMC5257026

[12] Willemink MJ Koszek WA Hardell C Wu J Fleischmann D. Preparing medical imaging data for machine learning. Radiology. (2020) 295:4–15. doi: 10.1148/radiol.2020192224, PMID: 32068507 PMC7104701

[13] Sowrirajan H Yang J Ng AY Rajpurkar P (2021) Moco pretraining improves representation and transferability of chest x-ray models. International Conference on Medical Imaging with deep learning (MIDL).

[14] Shen D Wu G Suk H-I. Deep learning in medical image analysis. Annu Rev Biomed Eng. (2017) 19:221–48. doi: 10.1146/annurev-bioeng-071516-044442, PMID: 28301734 PMC5479722

[15] Chen T Kornblith S Norouzi M Hinton G (2020) A simple framework for contrastive learning of visual representations International conference on machine learning (ICML).

[16] Wu Z Xiong Y Yu SX Lin D (2018) Unsupervised feature learning via non-parametric instance discrimination. IEEE/CVF conference on computer vision and pattern recognition (CVPR).

[17] Chen T Kornblith S Swersky K Norouzi M Hinton GE (2020) Big self-supervised models are strong semi-supervised learners. Annual conference on neural information processing systems (Neur IPS).

[18] He K Fan H Wu Y Xie S Girshick R (2020) Momentum contrast for unsupervised visual representation learning. IEEE/CVF Conference on Computer Vision and Pattern Recognition (CVPR).

[19] Chen X Fan H Girshick R He K. Improved baselines with momentum contrastive learning. ar Xiv. (2020). doi: 10.48550/arXiv.2003.04297

[20] Vaswani A Shazeer N Parmar N Uszkoreit J Jones L Gomez AN. Attention is all you need. Neural Inform Process Syst. (2017) 30:5998–08.

[21] Soares E Angelov P Biaso S Froes MH Abe DK. SARS-CoV-2 CT-scan dataset: a large dataset of real patients CT scans for SARS-CoV-2 identification. Med Rxiv. (2020) 10:1–8.

[22] Mei X Lee H-C Diao K-y Huang M Lin B Liu C. Artificial intelligence–enabled rapid diagnosis of patients with COVID-19. Nat Med. (2020) 26:1224–8. doi: 10.1038/s41591-020-0931-3, PMID: 32427924 PMC7446729

[23] He K Zhang X Ren S Sun J (2016) Deep residual learning for image recognition. IEEE/CVF Conference Computing Vision Pattern Recognisition.

[24] Chen J Wu L Zhang J Zhang L Gong D Zhao Y. Deep learning-based model for detecting 2019 novel coronavirus pneumonia on high-resolution computed tomography. Sci Rep. (2020) 10:19196–11. doi: 10.1038/s41598-020-76282-0, PMID: 33154542 PMC7645624

[25] Qiu Y Liu Y Li S Xu J. Miniseg: an extremely minimum network for efficient covid-19 segmentation. In: Proceedings of the AAAI Conference on Artificial Intelligence. Virtually: AAAI (2021).

[26] Wang G Liu X Li C Xu Z Ruan J Zhu H. A noise-robust framework for automatic segmentation of COVID-19 pneumonia lesions from CT images. IEEE Trans Med Imaging. (2020) 39:2653–63. doi: 10.1109/TMI.2020.3000314, PMID: 32730215 PMC8544954

[27] Panwar H Gupta P Siddiqui MK Morales-Menendez R Bhardwaj P Singh VJC. A deep learning and grad-CAM based color visualization approach for fast detection of COVID-19 cases using chest X-ray and CT-scan images. Chaos Solitons Fractals. (2020) 140:110190. doi: 10.1016/j.chaos.2020.110190, PMID: 32836918 PMC7413068

[28] Ma X Zheng B Zhu Y Yu F Zhang R Chen B. COVID-19 lesion discrimination and localization network based on multi-receptive field attention module on CT images. Optik. (2021) 241:167100. doi: 10.1016/j.ijleo.2021.167100, PMID: 33976457 PMC8103744

[29] Yang P Yin X Lu H Hu Z Zhang X Jiang R. CS-CO: a hybrid self-supervised visual representation learning method for H & E-stained histopathological images. Med Image Anal. (2022) 81:102539. doi: 10.1016/j.media.2022.102539, PMID: 35926337

[30] Zhang Y Jiang H Miura Y Manning CD Langlotz CP. Contrastive learning of medical visual representations from paired images and text. ar Xiv. (2020) 2010:2–25. doi: 10.48550/arXiv.2010.00747

[31] Chaitanya K Erdil E Karani N Konukoglu E. Contrastive learning of global and local features for medical image segmentation with limited annotations. Adv Neural Inf Proces Syst. (2020) 33:12546–58. doi: 10.48550/arXiv.2006.10511

[32] Zeng D Wu Y Hu X Xu X Yuan H Huang M. Positional contrastive learning for volumetric medical image segmentation. International Conference on Medical Image Computing and Computer-assisted Intervention. Strasbourg: Springer (2021).

[33] Wu Y Zeng D Wang Z Shi Y Hu J. Distributed contrastive learning for medical image segmentation. Med Image Anal. (2022) 81:102564. doi: 10.1016/j.media.2022.102564 35994968

[34] Khalifa NE Loey M Mirjalili S. A comprehensive survey of recent trends in deep learning for digital images augmentation. Artif Intell Rev. (2022) 55:2351–77. doi: 10.1007/s10462-021-10066-4, PMID: 34511694 PMC8418460

[35] Boyat AK Joshi BK. A review paper: noise models in digital image processing. Sig Image Process. (2015) 6:63. doi: 10.48550/arXiv.1505.03489

[36] Evangelista RC Salvadeo DH Mascarenhas ND. A new bayesian Poisson denoising algorithm based on nonlocal means and stochastic distances. Pattern Recog Lett. (2022) 122:108363. doi: 10.1016/j.patcog.2021.108363

[37] Zhuang T Leng S Nett BE Chen G-H. Fan-beam and cone-beam image reconstruction via filtering the backprojection image of differentiated projection data. Phys Med Biol. (2004) 49:5489–503. doi: 10.1088/0031-9155/49/24/007, PMID: 15724538

[38] Stierstorfer K Rauscher A Boese J Bruder H Schaller S Flohr T. Weighted FBP—a simple approximate 3D FBP algorithm for multislice spiral CT with good dose usage for arbitrary pitch. Phys Med Biol. (2004) 49:2209–18. doi: 10.1088/0031-9155/49/11/007, PMID: 15248573

[39] Huang J Ling CX. Using AUC and accuracy in evaluating learning algorithms. IEEE Trans Knowl Data Eng. (2005) 17:299–10. doi: 10.1109/TKDE.2005.50

[40] Barber JA Thompson SG. Analysis of cost data in randomized trials: an application of the non-parametric bootstrap. Stat Med. (2000) 19:3219–36. PMID: 11113956 10.1002/1097-0258(20001215)19:23<3219::aid-sim623>3.0.co;2-p

[41] Simonyan K Zisserman A. Very deep convolutional networks for large-scale image recognition. Ar xiv preprint ar xiv: 409.1556. (2014).

[42] Huang G Liu Z Van Der Maaten L Weinberger KQ (2017). Densely connected convolutional networks. IEEE/CVF conference on computer vision and pattern recognition (CVPR).

[43] Deng J Dong W Socher R Li L-J Li K Fei-Fei L. Imagenet: a large-scale hierarchical image database. In 2009 IEEE Conference on Computer Vision and Pattern Recognition. Miami: IEEE (2009).

[44] Gaur P Malaviya V Gupta A Bhatia G Pachori RB Sharma D. COVID-19 disease identification from chest CT images using empirical wavelet transformation and transfer learning. Biomed Sig Process Control. (2022) 71:103076. doi: 10.1016/j.bspc.2021.103076, PMID: 34457034 PMC8384584

[45] Ewen N Khan N (2021) Targeted self supervision for classification on a small covid-19 ct scan dataset. International symposium on biomedical imaging (ISBI).

[46] Yang X He X Zhao J Zhang Y Zhang S Xie P. COVID-CT-dataset: a CT scan dataset about COVID-19. Ar xiv preprint ar xiv: 2003.13865. (2020).

[47] Ahmed SAA Yavuz MC Şen MU Gülşen F Tutar O Korkmazer B. Comparison and ensemble of 2D and 3D approaches for COVID-19 detection in CT images. Neurocomputing. (2022) 488:457–69. doi: 10.1016/j.neucom.2022.02.018, PMID: 35345875 PMC8942080

[48] Chaudhary PK Pachori RB. FBSED based automatic diagnosis of COVID-19 using X-ray and CT images. Comp Biol Med Glob Surv. (2021) 134:104454. doi: 10.1016/j.compbiomed.2021.104454, PMID: 33965836 PMC8088544

[49] Wang Z Liu Q Dou Q. Contrastive cross-site learning with redesigned net for COVID-19 CT classification. IEEE J Biomed Health Inform. (2020) 24:2806–13. doi: 10.48550/arXiv.2009.07652, PMID: 32915751 PMC8545175

[50] Patel RK Kashyap M. Automated diagnosis of COVID stages from lung CT images using statistical features in 2-dimensional flexible analytic wavelet transform. Biocybernet Biomed Eng. (2022) 42:829–41. doi: 10.1016/j.bbe.2022.06.005, PMID: 35791429 PMC9247116

[51] Liu Z Lin Y Cao Y Hu H Wei Y Zhang Z. (2021) Swin transformer: hierarchical vision transformer using shifted windows. IEEE/CVF Conference on Computer Vision and Pattern Recognition (CVPR).

[52] Selvaraju RR Cogswell M Das A Vedantam R Parikh D Batra D Grad-cam: visual explanations from deep networks via gradient-based localization. IEEE/CVF International Conference on Computer Vision (ICCV) (2017).

